# Identification of cancer stem cell-related genes through single cells and machine learning for predicting prostate cancer prognosis and immunotherapy

**DOI:** 10.3389/fimmu.2024.1464698

**Published:** 2024-08-29

**Authors:** YaXuan Wang, Li Ma, Jiaxin He, HaiJuan Gu, HaiXia Zhu

**Affiliations:** ^1^ Cancer Research Centre Nantong, Affiliated Tumor Hospital of Nantong University, Nantong, China; ^2^ Department of Urology, The First Affiliated Hospital of Harbin Medical University, Harbin, China; ^3^ Department of Pharmacy, Tongren Hospital Affiliated to Wuhan University (The Third Hospital of Wuhan), Wuhan, China

**Keywords:** cancer stem cell, prostate adenocarcinoma, single cell analysis, machine learning, HSPE1

## Abstract

**Background:**

Cancer stem cells (CSCs) are a subset of cells within tumors that possess the unique ability to self-renew and give rise to diverse tumor cells. These cells are crucial in driving tumor metastasis, recurrence, and resistance to treatment. The objective of this study was to pinpoint the essential regulatory genes associated with CSCs in prostate adenocarcinoma (PRAD) and assess their potential significance in the diagnosis, prognosis, and immunotherapy of patients with PRAD.

**Method:**

The study utilized single-cell analysis techniques to identify stem cell-related genes and evaluate their significance in relation to patient prognosis and immunotherapy in PRAD through cluster analysis. By utilizing diverse datasets and employing various machine learning methods for clustering, diagnostic models for PRAD were developed and validated. The random forest algorithm pinpointed HSPE1 as the most crucial prognostic gene among the stem cell-related genes. Furthermore, the study delved into the association between HSPE1 and immune infiltration, and employed molecular docking to investigate the relationship between HSPE1 and its associated compounds. Immunofluorescence staining analysis of 60 PRAD tissue samples confirmed the expression of HSPE1 and its correlation with patient prognosis in PRAD.

**Result:**

This study identified 15 crucial stem cell-related genes through single-cell analysis, highlighting their importance in diagnosing, prognosticating, and potentially treating PRAD patients. HSPE1 was specifically linked to PRAD prognosis and response to immunotherapy, with experimental data supporting its upregulation in PRAD and association with poorer prognosis.

**Conclusion:**

Overall, our findings underscore the significant role of stem cell-related genes in PRAD and unveil HSPE1 as a novel target related to stem cell.

## 1 Introduction

Prostate adenocarcinoma (PRAD) ranks as the second most prevalent form of cancer and stands as the fifth highest contributor to cancer-related mortality among males across the globe (1, 2). Patients often lack significant clinical symptoms in the early stages, leading to advanced disease at diagnosis. Late-stage diagnosis results in missed treatment opportunities. While early-stage PRAD generally carries a good prognosis, outcomes worsen when patients progress to castration-resistant stages or develop metastasis. The introduction of targeted drugs like abiraterone acetate, bicalutamide, and enzalutamide has significantly improved the prognosis for PRAD patients (3). However, patient responses to drug treatments for PRAD vary, and there is a lack of specific markers for diagnosing the disease (4). While PSA is widely used as a serum marker for PRAD, its specificity and sensitivity have limitations. Recent advancements in PSA testing, including the use of indicators like free PSA and the free PSA/total PSA ratio, have enhanced the screening effectiveness for PRAD. Despite these improvements, the overall impact remains unsatisfactory. This underscores the critical need for identifying new diagnostic and prognostic markers to enhance outcomes for PRAD patients.

Cancer stem cells (CSCs) are a unique cell population that possesses the ability to self-renew and acquire diverse mutations over time, leading to resistance to cancer treatments, metastasis, and recurrence. Essentially, CSCs sustain tumor cell populations by continuously renewing themselves and proliferating limitlessly, while their migration capabilities contribute to tumor metastasis. These cells can remain inactive for extended periods and harbor various drug-resistant molecules, making them impervious to external factors that typically eradicate tumor cells. Consequently, even after conventional treatments eliminate the majority of tumor cells, relapse is still likely. PRAD Stem Cells (PCSC), along with prostate progenitor cells or prostate initiating cells, are present in PRAD and facilitate the progression of the disease (5). Growing evidence suggests that they are involved in the initiation, advancement, and response to androgen receptor-targeted therapies in PRAD, contributing to treatment resistance (6). The most recent study has revealed that berbamine is effective in targeting cancer stem cells and reversing cabazitaxel resistance in PRAD by inhibiting IGF2BP1 and p-STAT3 (7). Additionally, RCC2 has been found to promote proliferation and migration of PRAD cells through the Hh/GLI1 signaling pathway and cancer stem-like cells (8). Moreover, Lupeol, an inhibitor of the androgen receptor, has been shown to improve the sensitivity of PRAD stem cells to treatment with the anti-androgen enzalutamide (9). The significance of understanding the stemness characteristics of tumors in PRAD cannot be overstated, as it holds great promise for addressing clinical challenges associated with PRAD.

Single-cell RNA sequencing (scRNA-seq) represents a revolutionary technology that has markedly improved our understanding of the variety and behavior of cellular transcriptomes in various organisms (10). Numerous studies have illustrated the heterogeneity present in various tumor tissues, such as PRAD, through the use of scRNA-seq. Lai and Xu et al. identified CAFs-related genes using single-cell analysis and developed an online tool to predict clinical outcomes and radiotherapy prognosis for PRAD (11). Fan et al. demonstrated through single-cell sequencing analysis that the loss of AR-regulated AFF3 contributes to prostate cancer progression and decreases sensitivity to ferroptosis by downregulating ACSL4 (12). Cheng et al. revealed that autocrine IL11 mediates docetaxel resistance in prostate cancer by activating the JAK1/STAT4 pathway, as shown through single-cell deconvolution algorithm analysis (13). Single-cell analysis holds significant promise in the medical field by offering novel perspectives and tools for basic research, as well as making a substantial impact on clinical applications (14–16). With the ongoing technological advancements, single-cell analysis is anticipated to have a greater role in precision medicine, understanding disease mechanisms, and drug development. Through the analysis of clinical data and medical images from patients, machine learning algorithms can assist healthcare professionals in swiftly and accurately diagnosing diseases (17). In conclusion, the integration of single-cell analysis and machine learning techniques is becoming more prevalent in the medical field and is proving to be crucial. Our own research focused on identifying signature genes among stem cell markers through single-cell analysis, examining their correlation with prognosis, diagnosis, and immunotherapy in PRAD. By categorizing PRAD patients based on stem cell-related genes and analyzing their prognosis and response to immunotherapy using NMF clustering, we constructed an optimal diagnostic model that combines machine learning techniques and multiple datasets for validation. The core significance of HSPE1 was determined through the Random Forest (RF) algorithm and Friend analysis. The role of HSPE1 in PRAD was investigated using Gene Ontology (GO) and Kyoto Encyclopedia of Genes and Genomes (KEGG) analyses, with experimental validation confirming its expression and prognostic significance in this context. In summary, our research provides novel perspectives on potential markers and therapeutic targets for the diagnosis, prognosis, and immunotherapy of patients with PRAD.

## 2 Materials and methods

### 2.1 Datasets and patient samples

Four PRAD samples from the GSE168668 dataset were analyzed at the single-cell level. Additionally, RNAseq data and clinical details from the TCGA database’s prostate adenocarcinoma (PRAD) dataset were incorporated into the study. Various datasets, including TCGA-PRAD, GSE6956, GSE16120, GSE14206, and GSE32571, were used to create and validate diagnostic models. Sixty PRAD tissue samples, along with their adjacent tissue counterparts, were obtained from Shanghai Aoduo Biotechnology Company. The tissue chip study involved individuals who underwent surgical procedures between January 2011 and December 2014, with follow-up extending until November 2021, covering a period ranging from 6 to 10 years.

### 2.2 Processing of single-cell RNA-seq data

Four PRAD samples from the GSE168668 data set were utilized for single-cell analysis (18). Utilizing the Seurat package, we generated objects and filtered out poor-quality cells, ensuring that only high-quality data was included in our analysis. A standard data preprocessing procedure was then performed to examine the percentage of gene number, cell number, and mitochondrial content. The filtering criteria we used included genes detected in less than 3 cells and cells with fewer than 200 genes. Each cell was normalized by scaling the UMI count with a scale factor of 10,000, ensuring that the data was standardized and comparable across samples. After log transformation of the data, the Seurat (v3.0.2) ScaleData function was applied to further enhance the quality of the normalized data. The top 10 variable genes were selected for principal component analysis (PCA), allowing us to identify the key genes contributing to the variability in the dataset. We retained the first 11 principal components for UMAP visualization and clustering, providing insights into the underlying structure of the data. Cell clustering was performed using the FindClusters function within the Seurat R package, with a resolution set at 0.5 to ensure clear and distinct clustering patterns among the cells.

### 2.3 Negative matrix factorization cluster analysis and difference analysis in TCGA-PRAD dataset

The NMF algorithm is used to identify biologically significant coefficients in the gene expression matrix, organizing genes and samples to emphasize the internal structural characteristics of the data, which helps in grouping samples (19). Differential expression analysis comparing clusters A and B was performed using the ‘Limma’ R package with criteria of |logFC| > 0.5 and an adjusted p-value of <0.05. Subsequently, the ‘NMF’ R package was employed to cluster all samples based on the DEGs identified within the subclusters, aiming to unveil potential molecular subtypes. The ‘brunet’ algorithm with 100 iterations for each specified value and a range of 2 to 10 clusters was utilized. The optimal number of clusters was determined by considering cophenetic correlation, dispersion, and silhouette width (20). The Limma package in R software (version 3.40.2) was utilized to analyze the differential expression of mRNA between cancer and para-cancerous tissues in the TCGA-PRAD dataset.

### 2.4 Immune infiltration analysis

To ensure the credibility of the immune score results, we employed immunedeconv, an R software package (21). Thorough testing was conducted on each algorithm, revealing unique advantages. The selection of the XCELL method for this study was based on its ability to assess a wider range of immune cell types (22, 23).

### 2.5 Constructing diagnostic model

We combined multiple machine learning algorithms to create various algorithmic combinations aimed at developing diagnostic models related to PRAD. The algorithms employed include Random Forest (RF), Extreme Gradient Boosting (XGBoost), Elastic Net (Enet), Least Absolute Shrinkage and Selection Operator (Lasso), Ridge, Stepglm, glmBoost, Linear Discriminant Analysis (LDA), Gradient Boosting Machine (GBM), Support Vector Machine (SVM), and Naive Bayes. Training was conducted on the TCGA-PRAD dataset, with validation on the GSE6956, GSE16120, GSE14206, and GSE32571 datasets. Each combination was assessed based on its AUC value, and the best model was chosen based on the combination with the highest average AUC. The ROC curve analysis was conducted using the pROC [1.18.0] package, and the outcomes were visualized using ggplot2 [3.3.6].

### 2.6 Gene enrichment analysis

The study utilized GO to focus on molecular function (MF), biological pathways (BP), and cellular components (CC). KEGG Enrichment Analysis was used to explore gene functions and genome functional details. For further analysis of mRNA carcinogenesis, the ClusterProfiler package in R was utilized for GO function analysis of potential targets and KEGG pathway enrichment (24–26).

### 2.7 Expression and prognostic relevance of HSPE1 in PRAD tissue microarrays analyzed by immunofluorescence methods

First, immerse the paraffin sections in two tanks of xylene, soaking them for 15 minutes each. Subsequently, transfer the sections into absolute ethanol, followed by 95% ethanol, 85% ethanol, 75% ethanol, and distilled water, allowing 5 minutes for each solution. Upon completion of these steps, place the slices in a repair box containing pH 9.0 EDTA alkaline antigen repair solution and heat them in a pressure cooker for 2 minutes. After natural cooling, the sections should be placed in PBS (pH 7.4) and washed three times while shaking on a destaining shaker for 5 minutes each time. Next, immerse the slices in a 3% hydrogen peroxide solution and incubate at room temperature in the dark for 15 minutes. Following this, apply the blocking solution dropwise to ensure even coverage of the tissue, and allow it to block at room temperature for 30 minutes. Then, add the HSPE1 antibody (bs-7026R), diluted with antibody diluent, onto the sections and incubate overnight at 4°C. The next day, wash the sections three times with PBS for 5 minutes each time. After gently shaking the slices dry, add a poly-HRP secondary antibody corresponding to the species of the primary antibody dropwise, and incubate at room temperature in the dark for 10-20 minutes. The TSA fluorescent dye reaction solution should then be evenly applied to the sections and incubated at room temperature for 15 minutes. Afterward, apply DAPI ready-to-use dye on the sections and incubate at room temperature for 10 minutes in the dark. Finally, mount the slides and capture images under a fluorescence microscope. Immunostaining intensity was evaluated using a scale ranging from 0 to 3 to assess reaction strength, and another scale from 1 to 4 to determine the percentage of positive staining. The final expression score was calculated by multiplying the intensity score by the percentage scale score, yielding a total score that ranged from 0 to 5 for low expression, and from 6 to 12 for high expression (27).

### 2.8 Statistical analysis

The expression level of HSPE1 in both PRAD and normal tissues was assessed via the Wilcoxon rank-sum test. The log-rank test was utilized for conducting the prognostic analysis. Spearman correlation analysis was used to analyze the correlation between genes and stemness scores. A p-value of less than 0.05 was set as the threshold for statistical significance.

## 3 Result

### 3.1 Single-cell RNA-seq analysis and screening of stem cell-related marker genes

**Figure 1:**
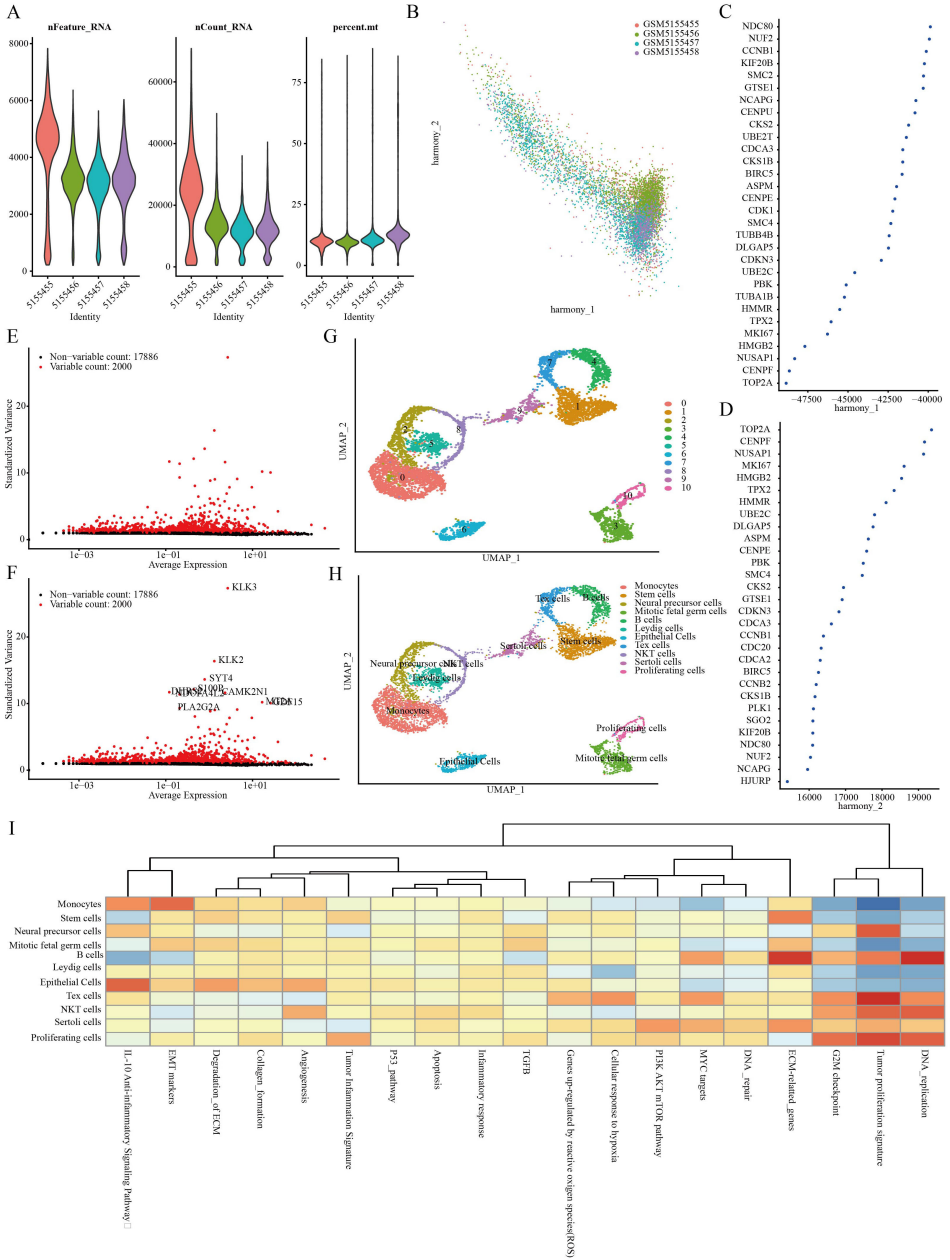
Identification of stem cell marker genes. **(A)** Quality control of scRNA-seq for cell sub-population. **(B–D)** Plot of PCA analysis after combined removal of batch effects. **(E, F)** Batch removal postcounts to find highly variable genes. **(G, H)** Stratification of PRAD samples by the umap method. **(I)** Functional analysis of different cell populations.

Four PRAD samples from the GSE168668 dataset were initially selected for single-cell analysis. Cell quality control criteria included a minimum of 200 RNAs per cell, a maximum of 5000 RNAs per cell, and a maximum of 10% mitochondrial RNAs per cell (**Figure 1A**). The filtered data underwent analysis using the HARMONY method focusing on highly variable genes, followed by batch removal analysis using these feature sets (**Figures 1B–D**). Variance analysis highlighted the top 10 genes that exhibited significant differential expression across cell samples, which include KLK3, KLK2, SYT4, S100P, and PLA2G2A (**Figures 1E, F**). The four PRAD samples were divided into 11 different cell groups through single-cell analysis, including Monocytes, Stem Cells, Neural Precursor Cells, Mitotic Fetal Germ Cells, B Cells, Leydig Cells, Epithelial Cells, Tex Cells, NKT Cells, Sertoli Cells, and Proliferating Cells (**Figures 1G, H**). Finally, an analysis of the function of these cell populations revealed that the stem cell populations were associated with extracellular matrix (ECM) related genes (**Figure 1I**).

### 3.2 Screening for stem cell-related prognostic differential genes

**Figure 2:**
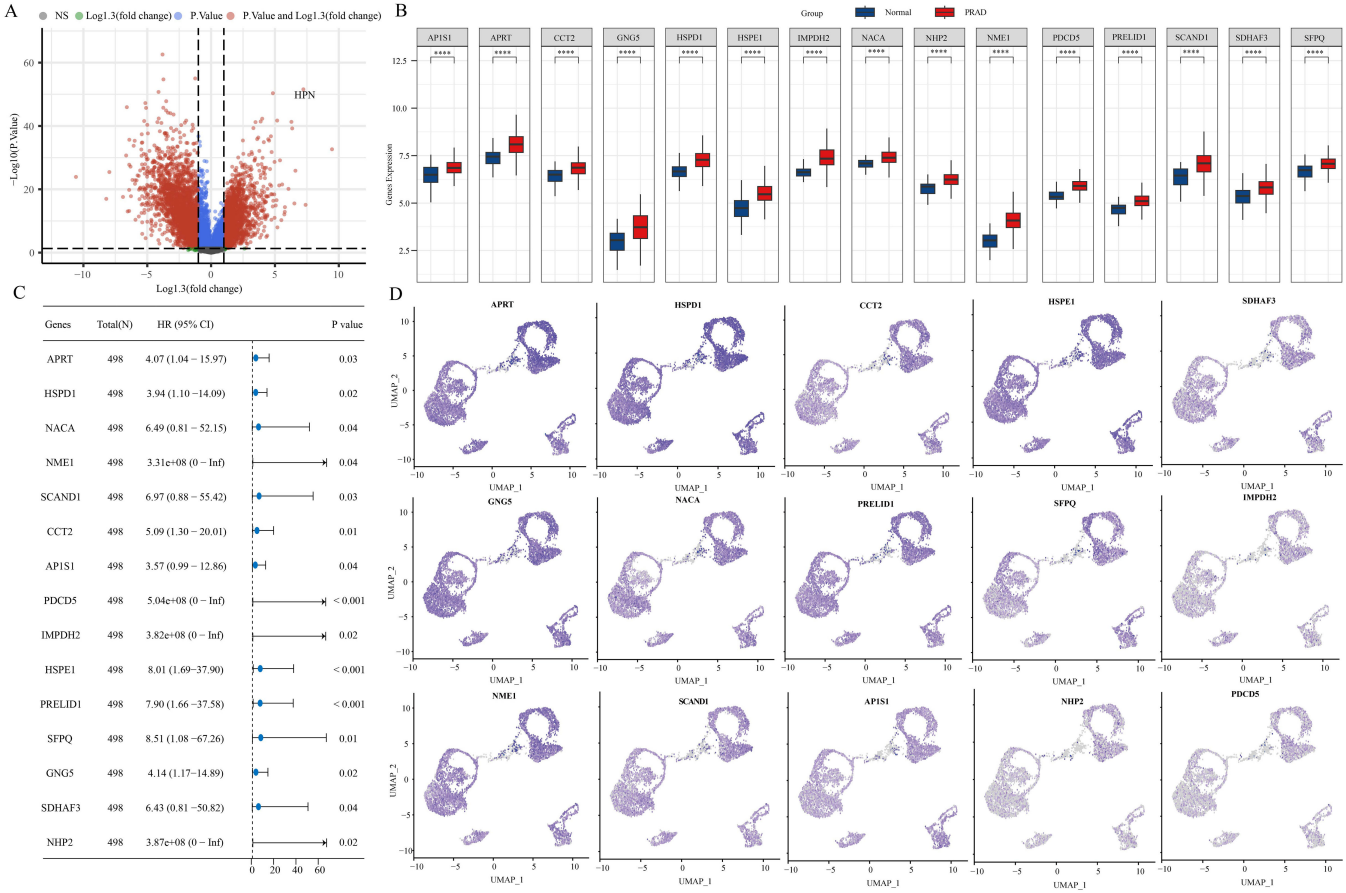
15 stem cell-related differential genes identified as associated with PRAD prognosis. **(A)** Variance analysis volcano chart. **(B)** Expression of stem cell-related differential genes. **(C)** Prognostic analysis of stem cell-associated differential genes. **(D)** Abundance of stem cell-associated differential genes in different cell populations. ****p< 0.0001.

Using ‘ P < 0.05 and Log2 (Fold Change) >1.3 or Log2(Fold Change) < −1.3’ as the criteria for differential analysis, 2110 genes exhibiting significantly increased expression in PRAD compared to normal prostate tissue were identified (**Figure 2A**). The 2110 genes that are highly expressed in PRAD were intersected with the stem cell-related genes identified previously and analyzed for their prognostic significance. Ultimately, 15 differential genes exhibiting stem cell characteristics related to PRAD prognosis were identified (**Figures 2B, C**). Further single-cell analysis showcased the abundance of these 15 genes within each cell population (**Figure 2D**).

### 3.3 Molecular typing based on stem cell-related genes

**Figure 3:**
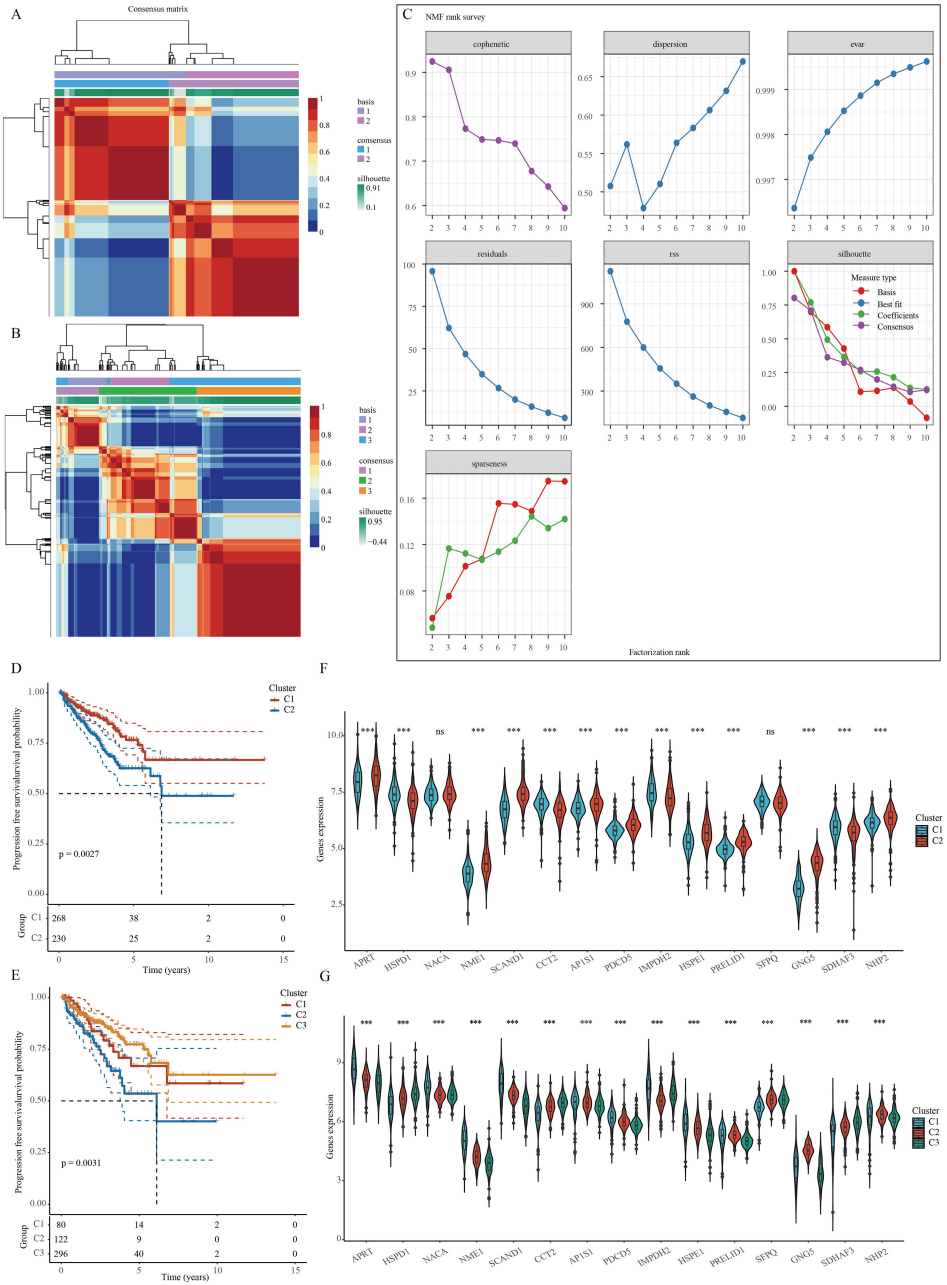
Clustering of PRAD samples based on NMF cluster analysis methods. **(A, B)** Consensus map of NMF clustering. **(C)** Assessment of performance and stability pertaining to clusters through multiple methods. **(D, E)** Survival differences between clusters. **(F, G)** Differences in the expression of stem cell-related genes between different clusters. ***p< 0.001; ns, not significant.

A NMF clustering algorithm was used to cluster the TCGA-PRAD samples. In order to determine the most suitable approach for dividing the TCGA-PRAD samples into subgroups for our subsequent studies, the current standard of judging based on the cophenetic curve is the clearest method. The optimal grouping is identified by the top point with the largest decrease in the cophenetic curve. Our study revealed that dividing the TCGA-PRAD samples into three groups according to the cophenetic curve is the most appropriate approach (**Figures 3A–C**). Analysis of the expression of specific genes related to stem cells was conducted across various groups. The results indicated a marked variance in gene expression among the groups, whether the TCGA-PRAD samples were categorized into 2 or 3 distinct groups. Moreover, when the samples were separated into 2 groups, individuals in cluster 1 showed a significantly superior prognosis in comparison to those in cluster 2. Conversely, when the samples were split into 3 groups, patients within cluster 3 displayed the most favorable prognosis while those in cluster 2 experienced the poorest prognosis. (**Figures 3D–G**).

### 3.4 Analysis of the correlation between stem cell-related genes and immunotherapy for PRAD

**Figure 4:**
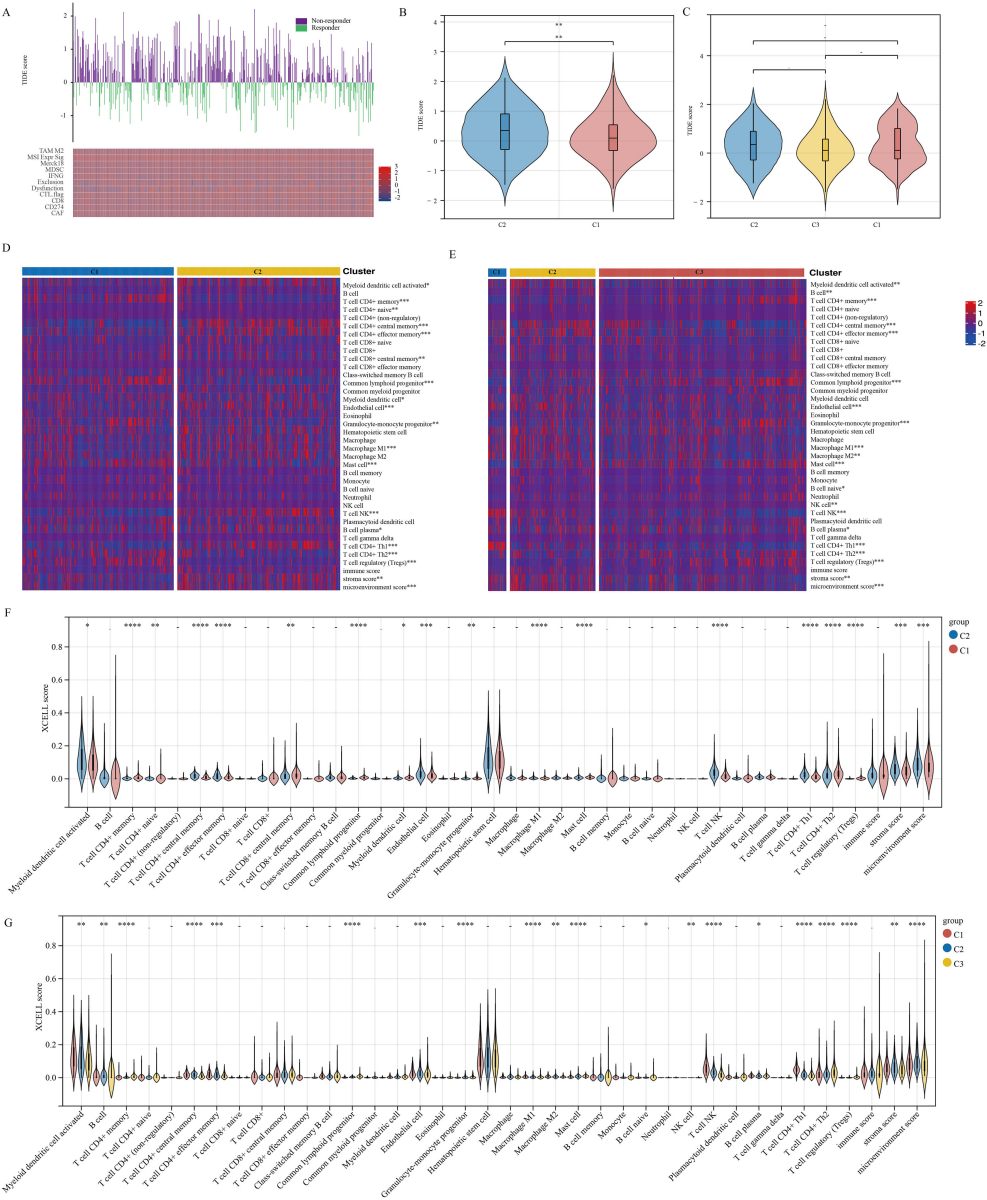
Stem cell marker genes are associated with immune infiltration in PRAD. **(A)** TIDE-based algorithm to assess responsiveness of TCGA-PRAD samples to immunotherapy. **(B, C)** Analysis of differences in TIDE scores between clusters. **(D, E)** Immune cell score heatmap. **(F, G)** Immune Cell Score Box Plot. *p< 0.05, **p< 0.01, ***p< 0.001,****p< 0.0001.

The therapy known as immune checkpoint blockade (ICB) has revolutionized cancer treatment in humans (28). For this research, we made use of the TIDE algorithm, which focuses on Tumor Immune Dysfunction and Exclusion, to forecast how effective immune checkpoint inhibitors will be for every specimen included in the TCGA-PRAD dataset (**Figure 4A**). The algorithm TIDE assesses two different ways of tumor immune avoidance, namely the impairment of cytotoxic T lymphocytes (CTLs) infiltrating the tumor and the resistance of CTLs to immunosuppressive elements. A high score of TIDE is linked to low effectiveness of ICB and decreased survival after ICB therapy. Upon dividing the TCGA-PRAD samples into two clusters, we observed a discrepancy in the response to ICB treatment between the clusters. However, this discrepancy was not evident when the samples were divided into three clusters (**Figures 4B, C**). xCell is a tool that evaluates the presence of immune cells by analyzing gene expression data in order to detect possible subgroups of immune cells and assess their proportion in tissues. In our study, we employed a specific algorithm to analyze variations in immune cell infiltration levels in TCGA-PRAD samples across different clusters. Our results suggest significant differences in various immune cell types, such as T cell CD4+ memory, T cell CD4+ central memory, T cell CD4+ effector memory, Common lymphoid progenitor, Endothelial cell, Macrophage M1, Mast cell, NKT cell, T cell CD4+ Th1, T cell CD4+ Th2, and Tregs, regardless of whether the samples were grouped into 2 or 3 clusters (**Figures 4D–G**).

### 3.5 Combination of machine learning algorithms to build diagnostic models

**Figure 5:**
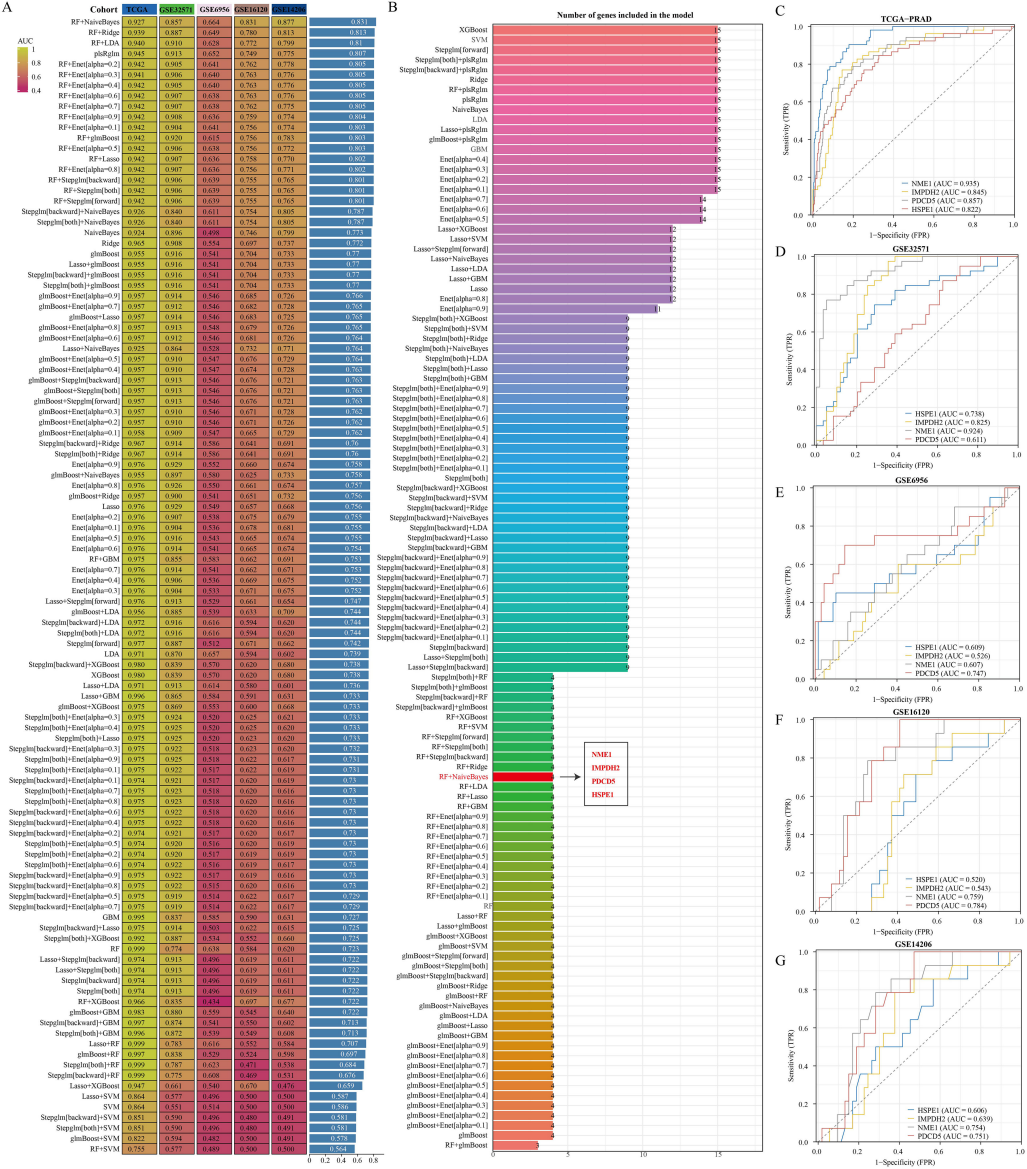
Identification of optimal diagnostic models based on machine learning algorithms. **(A)** Comparison of AUC values among diagnostic models created by various algorithm combinations. **(B)** Number of genes incorporated in diagnostic models built using different algorithm combinations. **(C–G)** Diagnostic significance of genes within diagnostic models across various datasets.

For early detection in PRAD patients, a diagnostic model centered on PRAD was developed. The training utilized the TCGA-PRAD dataset, while the validation involved four datasets: GSE6956, GSE32571, GSE16120, and GSE14206. Out of 108 tested algorithmic combinations, the RF+NaiveBayes pair proved to be the most effective for model construction (**Figure 5A**). The AUC value for the TCGA-PRAD training data was 0.927, and the corresponding AUC values for the validation datasets GSE6956, GSE32571, GSE16120, and GSE14206 were 0.857, 0.664, 0.831, and 0.877, respectively. The RF+NaiveBayes algorithm helped identify four critical genes: NME1, IMPDH2, PDCD5, and HSPE1 (**Figure 5B**). Additionally, ROC curves were plotted for these four genes across all the datasets, including TCGA-PRAD, GSE6956, GSE32571, GSE16120, and GSE14206 (**Figures 5C–G**).

### 3.6 HSPE1 identified as the most relevant gene among stem cell marker genes for PRAD prognosis and diagnosis

**Figure 6:**
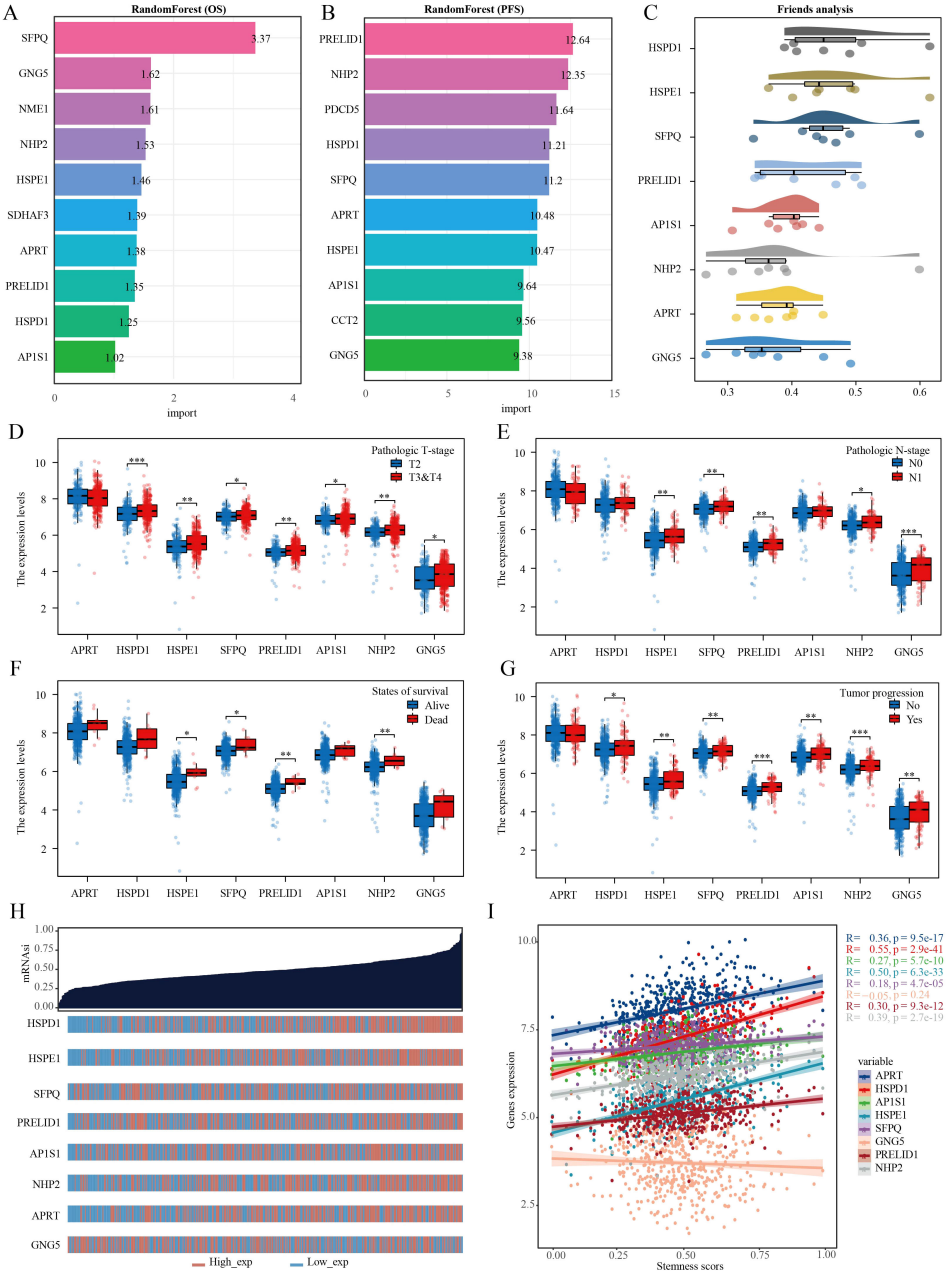
Multiple machine learning approaches identify genes most relevant to prognosis in PRAD. **(A)** Random forest algorithm identifies the top 10 genes most associated with OS in PRAD. **(B)** Random forest algorithm identifies the top 10 genes most associated with PFS in PRAD. **(C)** Friend analysis identifies key genes in stem cell-related genes. **(D–G)** Histogram of stem cell-related gene expression in different clinicopathologic parameters. **(H)** Dryness score and gene expression distribution map. **(I)** Correlation analysis of dryness score and gene expression. *p< 0.05, **p< 0.01, ***p< 0.001.

Utilizing the random forest algorithm, we conducted an analysis on stem cell-related genes in TCGA-PRAD samples to determine their association with patient overall survival (OS) and progression-free survival (PFS). HSPD1, HSPE1, SFPQ, PRELID1, AP1S1, NHP2, APRT, and GNG emerged as the top ten genes significantly linked to both OS and PFS in these patients (**Figures 6A, B**). The Friends analysis, a method that compares similarities between genes based on gene ontology, revealed HSPD1 and HSPE1 as the most crucial genes in this context (**Figure 6C**). We also discovered notable differences in the expression levels of HSPE1, SFPQ, PRELID1, and NHP2 when comparing various groups based on pathological stages, survival outcomes, and cancer progression status (**Figures 6D–G**). To quantify this, we employed the one-class logistic regression (OCLR) algorithm created by Malta et al. to calculate mRNAsi, a metric that represents the stemness of cells based on gene expression profiles. This analysis allowed us to gain insights into the molecular characteristics associated with cancer progression and prognosis based on the differential expression of these specific genes. Initially, we presented the expression profiles of stemness score and stem cell marker genes, followed by the calculation of correlation between stem cell marker genes and stemness score. Our findings indicated that HSPD1 and HSPE1 exhibited the strongest correlation with stemness score (**Figures 6H, I**). Considering these results and the genes incorporated in the diagnostic model, HSPE1 emerged as the most relevant and significant gene among stem cell marker genes for further investigation in PRAD progression.

### 3.7 Functional analysis of HSPE1 in PRAD

**Figure 7:**
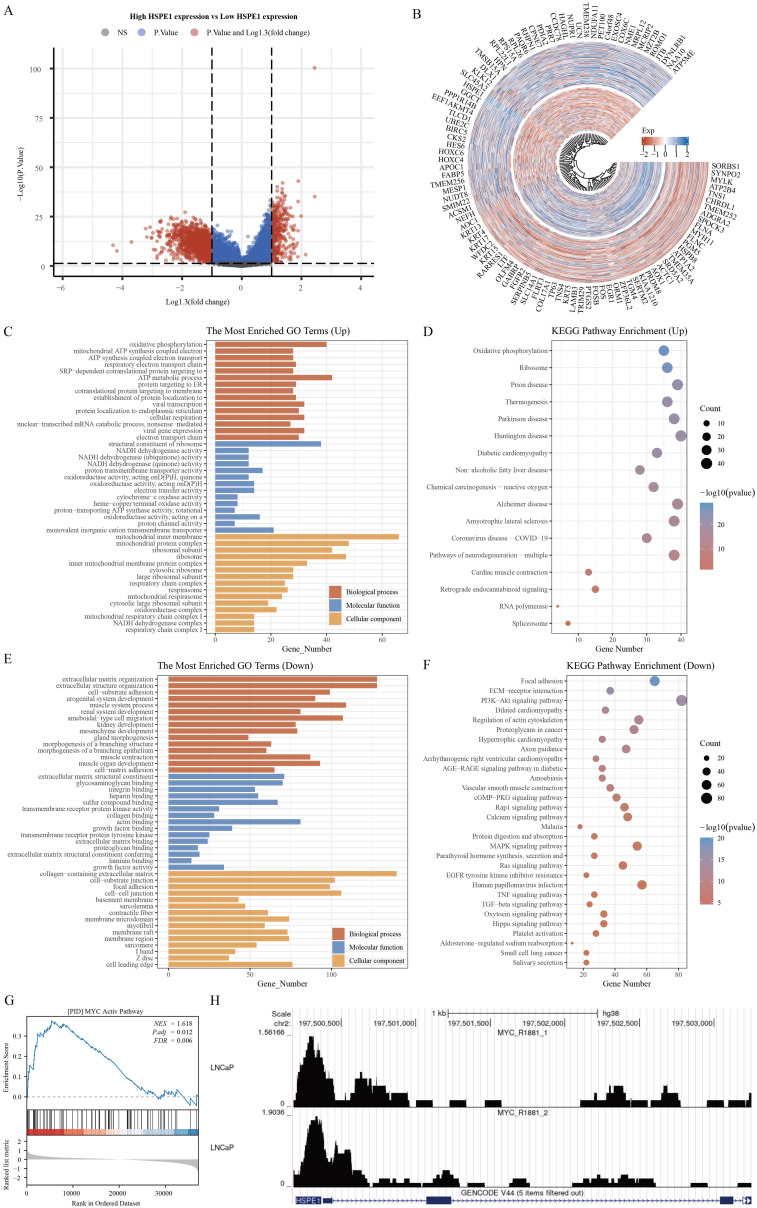
Functional analysis of HSPE1. **(A)** Variance analysis volcano map. **(B)** Differential Gene Expression Circle Map. **(C–F)** Functional analysis of HSPE1 in PRAD based on KEGG and GO methods. **(G)** Gene enrichment analysis results identify HSPE1 as associated with MYC activation in PRAD. **(H)** HSPE1 is associated with MYC transcriptional regulation in PRAD.

The samples in the TCGA-PRAD dataset were grouped according to the median expression levels of HSPE1. Samples exhibiting expression higher than the median were assigned to the high HSPE1 expression group, whereas those with lower expression were classified into the low HSPE1 expression group. Following this categorization, differential expression analysis was performed with P < 0.05 and Log2 (Fold Change) >1.3 or Log2(Fold Change) < −1.3 as the criteria for identifying significant differences (**Figures 7A, B**). The functions of HSPE1 were analyzed based on differential genes using GO. Among the upregulated genes, HSPE1 was found to be most related to the structural constituent of ribosome in the MF module, ATP metabolic process in the BP module, and mitochondrial inner membrane in the CC module. Among the genes exhibiting lower expression levels, HSPE1 showed strong correlation with actin binding in the MF category, organization of the extracellular matrix in the BP category, and collagen-containing extracellular matrix in the CC category. The utilization of KEGG enrichment analysis serves as an effective approach for dissecting gene functionalities and advanced genomic functional insights. Conversely, in the case of genes displaying higher expression levels, HSPE1 demonstrated predominant relevance to Huntington disease, while in contrast, among the genes displaying lower expression levels, HSPE1 was primarily associated with the PI3K-Akt signaling pathway (**Figures 7C–F**). GSEA results indicated a correlation between HSPE1 and the activation of MYC (**Figure 7G**). MYC, a widely recognized oncogene, has been linked to the advancement of PRAD. Given that MYC functions as a transcription factor, our analysis explored the possibility of a transcriptional regulatory association between HSPE1 and MYC, which was validated by our findings (**Figure 7H**). Consequently, we hypothesize that HSPE1 may promote oncogenesis by activating MYC.

### 3.8 Analysis of the correlation of immune infiltration of HSPE1 in PRAD

**Figure 8:**
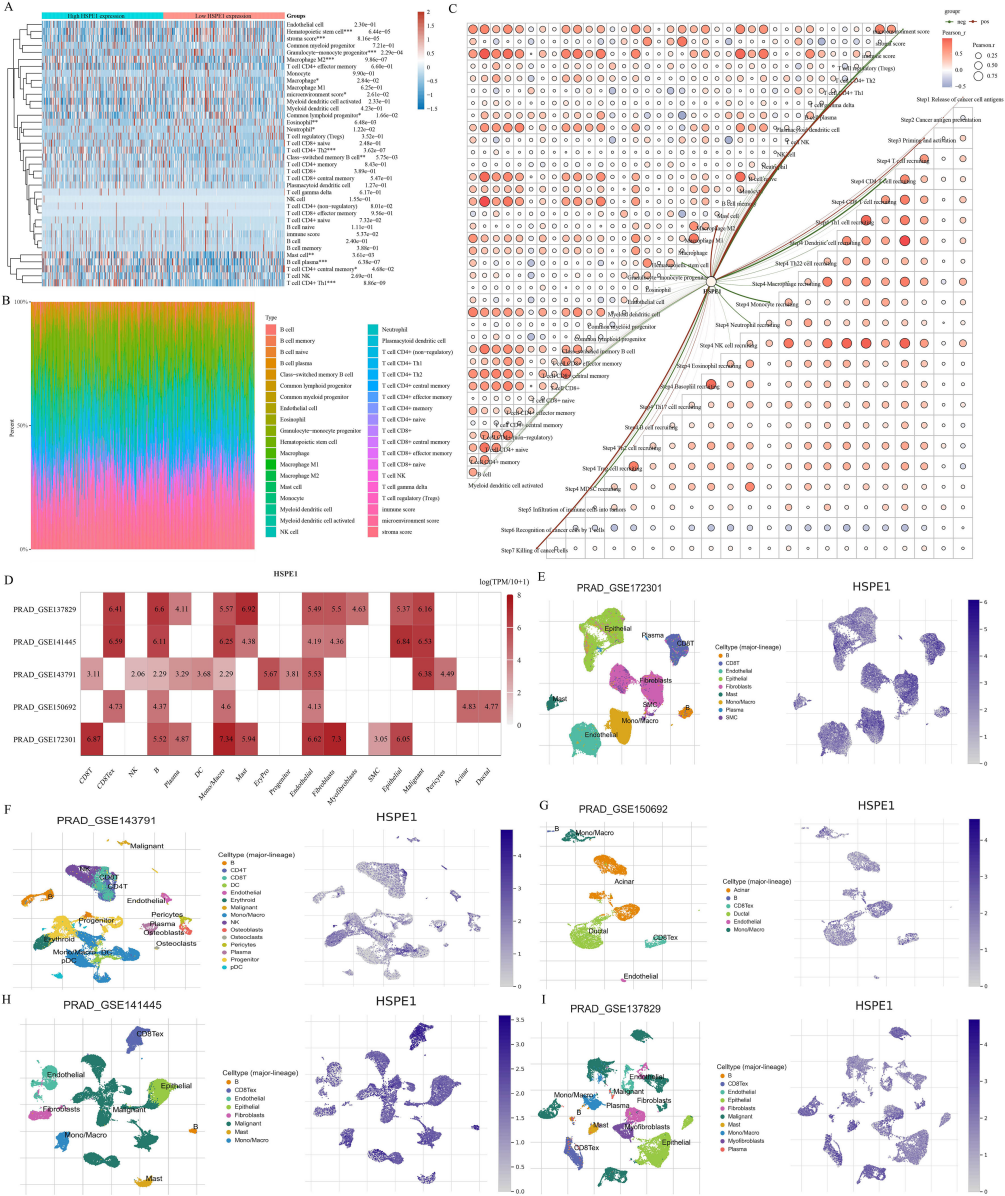
Investigating the relationship between HSPE1 expression and infiltration of immune cells. **(A)** Heatmap showing scores of immune cells. **(B)** Percentage of tumor-infiltrating immune cells in each sample. **(C)** Visual representation of the connection between HSPE1 expression and immune cell infiltration scores. **(D–I)** Analysis at the single-cell level revealing the link between HSPE1 expression and immune cell infiltration.

The TCGA-PRAD dataset samples were grouped based on HSPE1 expression to analyze differences in immune cell infiltration levels between the groups. Variations in B cell plasma, Mast cell, T cell CD4+ Th2, macrophage M2, and Granulocyte-monocyte progenitor infiltration levels were observed (**Figure 8A**). The distribution of immune cells infiltrating tumors in each TCGA-PRAD specimen was also illustrated (**Figure 8B**). Additionally, a correlation network diagram was generated to display the relationship between HSPE1 expression and scores of immune cell infiltration calculated using the XCELL algorithm and TIP algorithm, as well as the correlation analysis among the scores of different immune cells (**Figure 8C**). Finally, we also verified the above conclusions by single-cell analytical methods (**Figures 8D–I**).

### 3.9 Analysis of HSPE1-related targeted drugs

**Figure 9:**
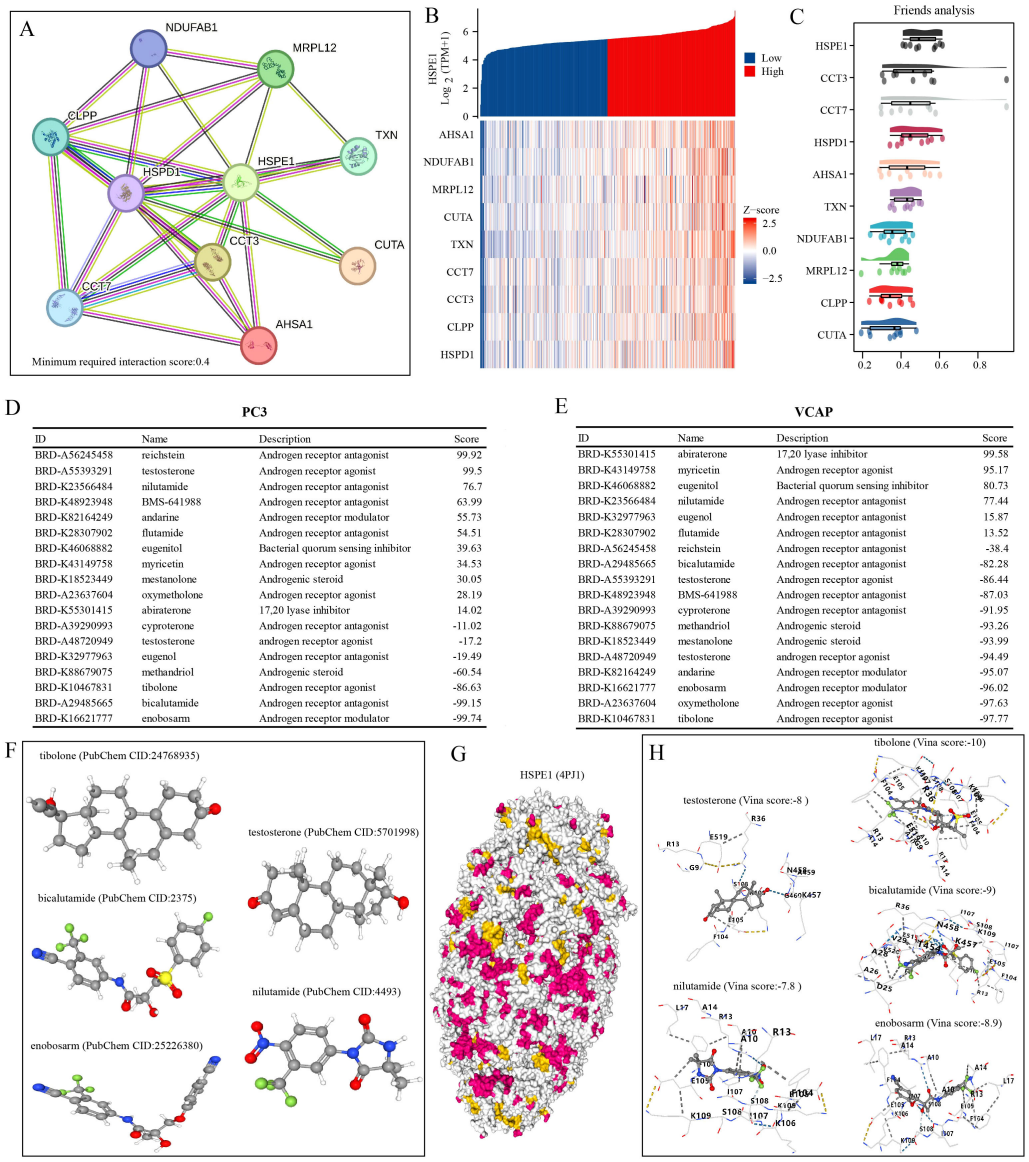
Identification of compounds with high HSPE1 relevance. **(A)** Network Diagram of HSPE1 Interacting Genes. **(B)** Heatmap of co-expression of HSPE1-interacting genes. **(C)** Similarity of HSPE1-related genes analyzed based on the Friends analysis method. **(D, E)** Analysis of HSPE1-related compounds based on the CMAP website. **(F–H)** Molecular docking of HSPE1 with HSPE1-related compounds.

To develop targeted drugs related to HSPE1, we initially identified 50 genes that interact with HSPE1 using the STRING website. Subsequently, we cross-referenced these genes with those positively associated with HSPE1 in the TCGA-PRAD dataset, resulting in the identification of 10 key genes that not only correlated with HSPE1 but also interacted with it (**Figures 9A, B**). Network analysis revealed the central role of HSPE1 among these genes (**Figure 9C**). Additionally, we analyzed the correlation of HSPE1-related genes with androgen-related compounds using the CMAP website, identifying five compounds with correlation scores exceeding 70 or falling below -70 in prostate cancer cells PC3 and VCAP (**Figures 9D, E**). To further verify the affinity of these compounds for HSPE1, we conducted molecular docking studies. It is widely accepted that a Vina score of less than -7 indicates a favorable docking effect. Our analysis demonstrates the strong binding affinity of these five compounds to HSPE1 (**Figures 9F–H**).

### 3.10 HSPE1 is highly expressed in PRAD and is associated with poor patient prognosis

**Figure 10:**
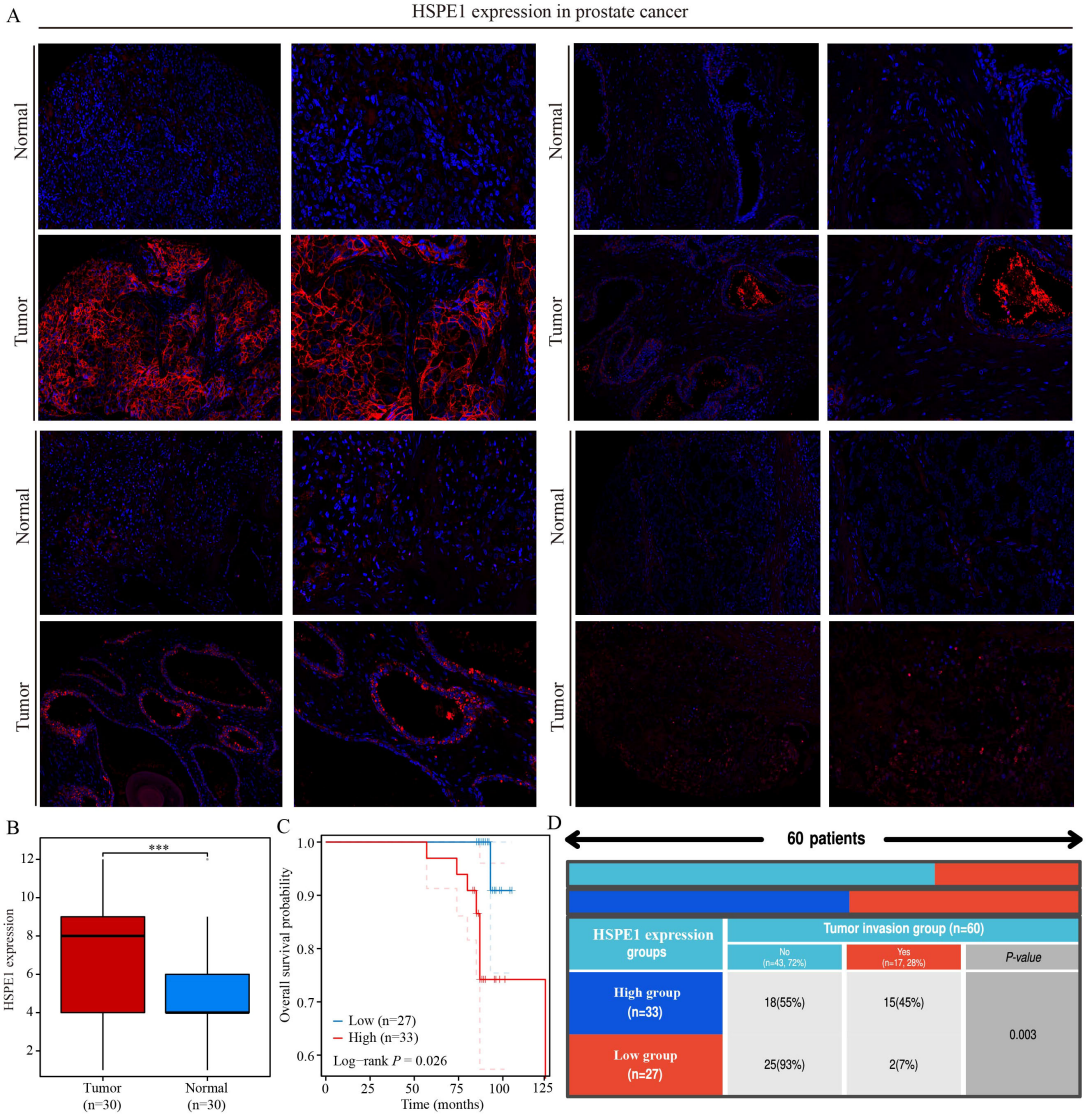
HSPE1 is highly expressed in PRAD and is associated with poor patient prognosis. **(A, B)** Differential expression of HSPE1 in PRAD. **(C)** KM curve of overall survival of HSPE1 in PRAD. **(D)** Analysis of the correlation between HSPE1 expression and tumor invasion. ***p< 0.001.

This study underscores the pivotal role of HSPE1 as a gene linked to PRAD metastasis. A total of 60 PRAD samples, along with corresponding normal prostate tissue samples, were collected, and immunofluorescence staining was conducted to investigate the differences in HSPE1 expression and its correlation with the prognosis of PRAD patients. The blue staining represents the cell nucleus, while the red staining indicates the expression of HSPE1. The findings demonstrated a notably higher HSPE1 expression in PRAD when compared to normal prostate tissue (**Figure 10A**). Furthermore, boxplots were employed to visually represent the variations in HSPE1 expression between PRAD and normal tissues (**Figure 10B**). Additionally, the analysis revealed a correlation between the expression of HSPE1 and the prognosis of PRAD patients, indicating that individuals with heightened levels of HSPE1 had a worse prognosis (**Figure 10C**). The tumors were classified based on their HSPE1 expression, and the relationship between tumor invasion and HSPE1 expression was evaluated. Remarkably, the occurrence of tumor invasion in the high-expression category of HSPE1 was notably higher than that in the low-expression category (**Figure 10D**).

## 4 Discussion

Analysis at the single-cell level can offer more precise and detailed cellular data, allowing for a deeper exploration of the dynamic changes and interactions among cells (29). By utilizing single-cell analysis, researchers can acquire a comprehensive understanding of cell functions, metabolism, signaling pathways, and other biological features, ultimately providing more precise information for disease diagnosis and treatment (30). PRAD is a prevalent malignancy affecting the male urinary tract, often diagnosed in advanced stages with metastasis (31, 32). Early detection is crucial for enhancing patient outcomes. In this study, we utilized single-cell analysis on 4 PRAD samples to identify stem cell marker genes. Furthermore, the advancement of tumors is closely associated with alterations in the tumor microenvironment, as tumor cells manipulate their surroundings by secreting different chemokines and cytokines (33–36). Our analysis also explored the correlation between stem cell marker genes and immune infiltration. Overall, our findings offer new insights into potential markers for early PRAD detection and improved patient prognosis.

In spite of notable progress in treatments that have enhanced the survival rates of cancer patients, the disease remains a leading global cause of death. Recent findings suggest that one significant reason many therapeutic approaches fail is their incapacity to eradicate stem cells, which are essential for initiating and sustaining tumor growth. Therefore, effectively targeting stem cells offers a hopeful strategy for managing cancer patients (37). In the context of single-cell analysis, 15 marker genes have been identified, some of which have established regulatory relationships with stem cells. For instance, an essential function in the self-renewal of embryonic stem cells and the viability of differentiated cells derived from them is carried out by the expression of HSPD1 (38). CCT2 has been found to sustain CSC properties and drive tumor advancement in epithelial ovarian cancer by inhibiting the proteasomal degradation of β-catenin (39). Additionally, the upregulation of SFPQ in extracellular vesicles derived from induced pluripotent stem cells has been demonstrated to safeguard retinal Müller cells from damage induced by hypoxia (40). The functional analysis of these cell populations revealed that the stem cell population is associated with ECM-related genes. There exists a significant interactive relationship between the ECM and CSCs, which plays a crucial role in the occurrence, development, and metastasis of tumors. For instance, the ECM provides a supportive microenvironment for CSCs, facilitating the maintenance of their stem cell properties, proliferation, and survival. Components of the ECM, such as collagen, fibronectin, and glycosaminoglycans, can activate signal transduction pathways and promote the self-renewal and differentiation of stem cells by interacting with receptors on the surface of CSCs (41). Cluster analysis was conducted on PRAD samples in the TCGA-PRAD dataset using the NMF algorithm based on the expression of 15 selected stem cell-related genes. The cophenetic curve suggested that dividing PRAD samples into 3 groups yielded the best grouping. Regardless of whether PRAD samples were divided into 3 groups or 2 groups, significant differences in patient prognosis were observed between the groups, with patients in cluster 2 consistently having the worst prognosis. To investigate the reasons behind these differences in patient prognosis, the response of patients to immune checkpoint inhibitor treatment among different clusters was analyzed using the TIDE algorithm. Results showed that patients in cluster 2 exhibited higher TIDE scores and poorer responses to immunotherapy, potentially contributing to the unfavorable prognosis observed in this cluster.

Due to the absence of clear diagnostic markers for PRAD patients, many individuals are unfortunately diagnosed at an advanced stage of the disease (42). To address this issue, we conducted a comprehensive follow-up analysis. Our study involved the development of a PRAD diagnostic model utilizing various machine learning algorithms. This model, which focused on the genes NME1, IMPDH2, PDCD5, and HSPE1, yielded promising results. In the training set, our model demonstrated strong performance, achieving an AUC value of 0.927. To further validate the effectiveness of our diagnostic model, we tested it against four different datasets. While one dataset showed suboptimal results, the remaining three datasets consistently confirmed the robustness and reliability of the diagnostic model we have constructed. The stemness score of each sample in PRAD samples was calculated using the OCLR algorithm developed by Malta et al. (43). Within the TCGA-PRAD dataset, out of the 15-stem cell-related marker genes that were chosen, only one gene’s expression did not show a significant correlation with the stemness score. This further validates the accuracy of the selected genes.

Utilizing the random forest algorithm, we identified the top 10 genes from a pool of 15 stem cell marker genes that exhibited associations with both OS and PFS in PRAD patients. By integrating the expression levels of these genes across various pathological stages, we pinpointed HSPE1 as a significant stem cell marker gene linked to the prognosis and progression of PRAD. KEGG functional analysis confirmed that HSPE1 exhibits the strongest correlation with the PI3k-Akt signaling pathway. This pathway, known as PI3K/Akt/mTOR, plays a crucial role in CSCs by regulating stemness, proliferation, differentiation, epithelial-to-mesenchymal transition, migration, and autophagy (44). Therefore, we inferred that HSPE1 may affect the stemness of PRAD cells by regulating the PI3k signaling pathway. The GSEA results indicated a robust correlation between HSPE1 and the transcription factor MYC. Additionally, our analysis revealed a significant enrichment of MYC in the HSPE1 promoter, thus confirming a direct regulatory relationship between HSPE1 and MYC. The role of the oncogene MYC in regulating stem cells in various tumors is well-established (45–49). It is highly probable that HSPE1 influences the stemness properties of PRAD by interacting with MYC. To develop targeted drugs related to HSPE1, we analyzed the correlation between HSPE1 and androgen receptor-related drugs using the CMAP website. Our findings indicate a significant relationship between HSPE1 and bicalutamide, a targeted drug commonly utilized in the clinical treatment of PRAD patients. Furthermore, the molecular docking results corroborate these findings. Molecular docking can predict potential interactions between drug molecules and their targets, thereby serving as a foundation for drug development. The results of docking studies yield critical insights into the mechanisms of drug action and are essential for a comprehensive understanding of how drugs influence cellular biological processes. Numerous studies have validated the significance of genes as drug targets through the application of molecular docking methods (50). Our results highlight the potential of HSPE1 as a candidate for PRAD-targeting drug development. In addition, we conducted immunofluorescence experiments to examine the expression levels and prognostic implications of HSPE1 in PRAD samples. The findings from our study further support the significance of HSPE1 in both the diagnosis and prognosis of PRAD. Our primary analyses utilized the TCGA-PRAD dataset. To enhance the robustness of our findings, it is crucial to include a larger sample size and a diverse validation set. Furthermore, additional experiments are necessary to confirm our conclusions.

## 5 Conclusion

Through single-cell analysis and utilization of multiple machine learning algorithms, our research has established a significant correlation between stem cell marker genes and the prognosis, diagnosis, and immune infiltration in patients with PRAD. Among these genes, HSPE1, identified as a key stem cell marker gene, emerges as particularly crucial in the progression of PRAD. Notably, HSPE1 exhibits a strong association with the diagnosis, prognosis, and immune infiltration patterns in PRAD patients. Furthermore, through experimental validation, the study underscores the pivotal role of HSPE1 in PRAD. Ultimately, our findings contribute valuable insights by introducing novel biomarkers and potential therapeutic targets for early detection of PRAD and enhancement of patient outcomes.

## Data Availability

The original contributions presented in the study are included in the article/supplementary material. Further inquiries can be directed to the corresponding authors.
